# Development of branchial ionocytes in embryonic and larval stages of cloudy catshark, *Scyliorhinus torazame*

**DOI:** 10.1007/s00441-024-03897-4

**Published:** 2024-05-15

**Authors:** Mayu Inokuchi, Yumiko Someya, Keitaro Endo, Katsunori Kamioka, Wataru Katano, Wataru Takagi, Yuki Honda, Nobuhiro Ogawa, Kazuko Koshiba-Takeuchi, Ritsuko Ohtani-Kaneko, Susumu Hyodo

**Affiliations:** 1 Department of Aquatic Bioscience, Graduate School of Agricultural and Life Sciences, The University of Tokyo, 1-1-1 Yayoi, Bunkyo, Tokyo 113-8657 Japan; 2 Department of Life Sciences, Toyo University, Itakura, Gunma 374-0193 Japan; 3 Laboratory of Physiology, Atmosphere and Ocean Research Institute, The University of Tokyo, Kashiwa, Chiba 277-8564 Japan

**Keywords:** Ionocyte, Elasmobranch, Embryo, Follicular structure, Ionoregulation

## Abstract

In teleost fish, branchial ionocytes are important sites for osmoregulation and acid-base regulation by maintaining ionic balance in the body fluid. During the early developmental stages before the formation of the gills, teleost ionocytes are localized in the yolk-sac membrane and body skin. By comparing with teleost fish, much less is known about ionocytes in developing embryos of elasmobranch fish. The present study investigated the development of ionocytes in the embryo and larva of cloudy catshark, *Scyliorhinus torazame*. We first observed ionocyte distribution by immunohistochemical staining with anti-Na^+^/K^+^-ATPase (NKA) and anti-vacuolar-type H^+^-ATPase (V-ATPase) antibodies. The NKA- and V-ATPase-rich ionocytes appeared as single cells in the gill filaments from stage 31, the stage of pre-hatching, while the ionocytes on the body skin and yolk-sac membrane were also observed. From stage 32, in addition to single ionocytes on the gill filaments, some outstanding follicular structures of NKA-immunoreactive cells were developed to fill the inter-filament region of the gill septa. The follicular ionocytes possess NKA in the basolateral membrane and Na^+^/H^+^ exchanger 3 in the apical membrane, indicating that they are involved in acid-base regulation like single NKA-rich ionocytes. Three-dimensional analysis and whole-mount immunohistochemistry revealed that the distribution of follicular ionocytes was limited to the rostral side of gill septum. The rostral sides of gill septum might be exposed to faster water flow than caudal side because the gills of sharks gently curved backward. This dissymmetric distribution of follicular ionocytes is considered to facilitate efficient body-fluid homeostasis of catshark embryo.

**Supplementary Information:**

The online version contains supplementary material available at 10.1007/s00441-024-03897-4.

## Introduction

Body fluid homeostasis is essential for optimal cell function. Fish have developed particular osmoregulatory and acid-base regulatory mechanisms and extended their habitats into diverse aquatic environments. Teleost fish, the majority of fish species, maintain their plasma osmolality within narrow physiological ranges, equivalent to one-third the osmolality of seawater (SW), as terrestrial vertebrates. It is well known that ionocytes in the gills are responsible for ion uptake and secretion against the concentration gradients imposed by freshwater (FW) and SW environments. The function of ionocytes is determined by the localization of various ion-transporting proteins in the apical and basolateral membranes. In teleost fish, active ion secretion of SW-type ionocyte is mediated by Na^+^/K^+^-ATPase (NKA) and Na^+^, K^+^, 2Cl^−^ cotransporter 1 (NKCC1, slc12a2) in the basolateral membrane, and by the Cl^−^ channel, cystic fibrosis transmembrane conductance regulator (CFTR), in the apical membrane (Hwang and Lin 2013). By contrast, the mechanisms for ion uptake in ionocytes of FW-acclimated teleosts vary according to species (Hwang and Lin 2013). In euryhaline Mozambique tilapia, two distinct types of ionocytes have been identified in FW: ionocytes with apical Na^+^, Cl^−^ cotransporter 2 (NCC2, slc12a10) and those with apical Na^+^/H^+^ exchanger 3 (NHE3, slc9a3) (Hiroi et al. 2008; Inokuchi et al. 2022). Stenohaline zebrafish, another model fish species for iono- and osmo-regulation study, has five types of ionocytes: vacuolar-type H^+^-ATPase (V-ATPase)-rich (HR) cell, NKA-rich (NaR) cell, NCC cell, solute carrier 26-expressing (slc26) cell, and K^+^ secreting (KS)-cells (Guh et al. 2015). In teleost fish embryos, before the formation of osmoregulatory organs, a rich population of ionocytes has been reported to be present in the yolk-sac membrane and body skin (Lasker and Threadgold 1968; Guggino 1980; Hwang and Hirano 1985). The occurrence of distinct FW- and SW-type ionocytes in the yolk-sac membrane was found in tilapia embryos, and the ionocytes alter their morphology and function within 3 days after transfer from FW to SW, similar to the case with branchial ionocytes in adult fish (Hiroi et al. 2008; Choi et al. 2011; Inokuchi et al. 2022).

In comparison with teleost fish and terrestrial vertebrates, cartilaginous fish adopt a unique strategy for body fluid homeostasis. Marine cartilaginous fish retain a high concentration of urea in their body fluid, which is isosmotic or slightly hyperosmotic with respect to surrounding SW (Smith 1931; Robertson 1989). Ionocytes have also been found in elasmobranch gill epithelia, but their function has been considered to be different from those of teleosts (Evans et al. 2005). Immunohistochemical observations of ion-transporting proteins have revealed that there are two types of ionocytes in the gills of the elasmobranch species studied so far: type-A ionocytes and type-B ionocytes in Atlantic stingray (*Dasyatis sabina*), spiny dogfish (*Squalus acanthias*), bull shark (*Carcharhinus leucas*), leopard shark (*Triakis semifasciata*), bamboo shark (*Chiloscyllium punctatum*), and houndshark (*Triakis scyllium*) (Piermarini and Evans 2001; Choe et al. 2005, 2007; Reilly et al. 2011; Roa et al. 2014; Cramp et al. 2015; Takabe et al. 2016). The type-A ionocytes possess NKA in the basolateral membrane and NHE3 in the apical membrane. On the other hand, type-B ionocytes co-express V-ATPase in the basolateral membrane and pendrin-like Cl^−^/HCO_3_^–^ exchanger (PDN, slc26a4) in the apical membrane (Piermarini et al. 2002). The vast majority of elasmobranchs reside in SW for their entire life, but the localization patterns of ion transporting proteins in elasmobranch ionocytes are similar to those of FW-type ion-absorptive ionocytes in teleost fish. In euryhaline bull shark, mRNA expressions of NHE3 and NKA was significantly upregulated in the gills of FW-captured bull shark relative to estuary/SW-captured fish, suggesting that type-A ionocytes may be important in branchial Na^+^ uptake in FW environments (Reilly et al. 2011). In stenohaline houndshark, acclimation to a low-salinity environment (30%-diluted SW) induced increases in the numbers of type-A and type-B cells (Takabe et al. 2016). These findings indicate that elasmobranch ionocytes contribute to hyper-osmoregulatory ability. Since these two types of ionocytes occupy a great portion of gill epithelia also in SW elasmobranchs, it is thought that their function is most likely acid-base regulation rather than NaCl excretion in SW environment. Instead of secretion from gill ionocytes in teleost fish, marine elasmobranchs secrete NaCl in the rectal gland, which expresses NKCC1 and CFTR similar to SW-type ionocytes of teleost fish (Burger 1965; Riordan et al. 1994).

The reproductive strategies of cartilaginous fish are diverse, and their reproduction is divided into two main modes, oviparity and viviparity. In oviparous species, eggs are laid within several weeks of fertilization, and the embryo develops inside the egg capsule for several months before hatching (Ballard et al. 1993; Rodda and Seymour 2008; Musa et al. 2018). The 34 successive developmental stages of cloudy catshark can be determined according to the table of developmental stages for the lesser spotted dogfish (Ballard et al. 1993; Takagi et al. 2017). It is widely accepted that the external gill is an important respiratory organ for elasmobranch embryos during the early to mid stages of development (Baranes and Wendling 1981; Hamlett et al. 1985). In catshark, the external gills appear at stage 27 and reach a maximum length at stage 32 (Takagi et al. 2017). Thereafter, the external gills regressed and disappeared by stage 34, whereas the gill lamellae developed and remained inside the gill slit (Takagi et al. 2017; Tomita et al. 2014).

At stage 31, after one-third of the developmental period has passed, the anterior part of the egg capsule opens, which is known as the “pre-hatching” (Ballard et al. 1993; Takagi et al. 2017; Honda et al. 2020). Because the embryos prior to pre-hatching are surrounded by a jelly-like substance in the closed egg case, the intracapsular fluid appears to be isolated from the external SW. Takagi et al. (2017) demonstrated that there was no difference in chloride concentrations between the intracapsular environment of catshark and the surrounding SW throughout development, regardless of whether the egg capsule was tightly closed (before the pre-hatching) or not (after the pre-hatching). This report indicated that embryo of oviparous elasmobranch needed to regulate internal homeostasis even before the pre-hatching event. In embryos of marine cartilaginous fish species, elephant fish, and cloudy catshark, the extraembryonic yolk-sac membrane contribute to urea production to maintain their body fluid osmolality (Takagi et al. 2014, 2017). However, the mechanisms of ion and acid-base regulation in cartilaginous fish during developmental period including the development of ionocytes remain to be elucidated.

In the present study, we aimed to clarify the functional development of ionocytes in oviparous cloudy catshark. We observed the ontogenic changes in the distribution of ionocytes and the localization of major ion-transporting proteins in ionocytes. After prehatching, we recognized that a follicular structure of ionocytes developed in the gill septum. Our morphological and molecular studies revealed that the follicular structure of ionocytes is distributed only in the rostral side of the gill septum and might contribute to the efficient body fluid homeostasis in catshark embryo.

## Materials and methods

### Animal

Spawned cloudy catshark (*Scyliorhinus torazame*) eggs collected from captive individuals were transported from Ibaraki Prefectural Oarai Aquarium to the Atmosphere and Ocean Research Institute at the University of Tokyo. They were kept in floating baskets in a 1000-L tank with recirculating natural SW under a constant photoperiod (12 h:12 h light:dark) at 16 °C. The developmental stages of cloudy catshark embryos were identified according to the developmental stages of lesser spotted dogfish and cloudy catshark (Ballard et al. 1993; Takagi et al. 2017). Once hatched, juveniles were maintained under similar environmental conditions and fed a diet of chopped squid twice a week ad libitum. All animal experiments were conducted according to the Guidelines for Care and Use of Animals approved by the ethics committee of the University of Tokyo (P19-2).

### Sampling

The embryos from developmental stage 30 to 34 and juveniles were anesthetized with 0.02% ethyl 3-aminobenzoate methanesulfonate (Sigma-Aldrich, St. Louis, MO). Their whole bodies were fixed in formalin with picric acid (saturated picricacid:formalin 3:1) or 4% paraformaldehyde (PFA) in 0.1 M sodium phosphate buffer (PB, pH 7.4) at 4 °C overnight, and stored in 70% ethanol.

### Antiserum

For immunocytochemical detection of NKA-immunoreactive A-type ionocytes, we used a rabbit polyclonal antiserum raised against a synthetic peptide corresponding to part of the highly conserved region of the NKA α-subunit (NAK121, a gift from Prof. T. Kaneko, the University of Tokyo), which has been widely used to detect branchial NKA in teleost fish (Uchida et al. 2000). To detect B-type ionocytes, a rabbit polyclonal antibody for V-ATPase was raised against the B subunit of eel (*Anguilla anguilla*) V-ATPase (see Wilson et al. 2007; Reis-Santos et al. 2008). The antibody against the same site recognized V-ATPase in the gills of bull shark and brown-banded bamboo shark (Reilly et al. 2011; Cramp et al. 2015). A polyclonal antibody was raised in a guinea pig against a synthetic peptide (LLADISEEHPLSFLPESSM) corresponding to the highly conserved C-terminal region of vertebrate NHE3 molecules (Choe et al. 2005). Anti-V-ATPase antisera were produced in a rabbit and a guinea pig, while anti-NHE3 antiserum was produced in a guinea pig by Protein Purify Co. Ltd. (Gunma, Japan).

### Light microscopy

After fixation, the head region of embryos and juveniles was dissected out and dehydrated in an ethanol series, cleared with xylene, and embedded in paraplast (Leica, Wetzlar, Germany). Serial cross sections (10 μm) were cut and mounted on glass slides (Matsunami Glass, Osaka, Japan). To observe the development of the gills, sections of embryos from developmental stage 30–34 and juveniles were stained with hematoxylin and eosin (HE, Sakura Finetek Japan, Tokyo, Japan). Another section was then double immunohistochemically stained with the antisera specific for NKA and V-ATPase by the avidin-biotin-peroxidase complex (ABC) method using commercial reagents (Vectastain ABC kit, Vector Laboratories, Burlingame, CA). Briefly, deparaffined sections were incubated with 0.6% H_2_O_2_ for 30 min to block endogenous peroxidase activity and treated with 2% normal goat serum (NGS) in 0.01 M phosphate-buffered saline (PBS, pH7.4) for 30 min to reduce nonspecific staining. The anti-NKA antibody, diluted 1:2000 with PBS containing 2% NGS, was applied to the sections and incubated overnight at 4 °C. Subsequently, the sections were incubated with biotinylated anti-rabbit IgG for 30 min, and then with ABC for 60 min. The final reaction product was visualized using 0.02% 3,3′-diaminobenzidine tetrahydrochloride (DAB) in 0.05 M Tris–HCl buffer (pH 7.6) containing 0.005% H_2_O_2_. The brown DAB precipitation was detected on the gills, and the slides were rinsed in deionized H_2_O to stop the reaction. For double immunohistochemical staining of the same sections, the sections were incubated with 0.6% H_2_O_2_ for 30 min again. After blocking with 2% NGS in PBS for 30 min, the antibody to V-ATPase (polyclonal antibody serum diluted 1:5000) was then applied to the sections and incubated overnight at 4 °C. Rinsing and developing precipitation were performed as described above, except that a blue chromogen was used (Vector SG, Vector Laboratories). The sections were observed under a light microscope (Eclipse Ci-L, Nikon, Tokyo, Japan) and micrographs were obtained using a digital camera (MC-170HD; Leica).

### Confocal laser scanning microscopy

For observation of follicular ionocytes, embryos of stages 32 or 33 were examined. Deparaffined sections were incubated sequentially with 2% NGS in PBS for 30 min, a mixture of the primary antibodies diluted with 2% NGS in PBS overnight at 4 °C and a mixture of the secondary antibodies for 2 h at room temperature. For first double immunofluorescence staining, anti-NKA antiserum (1:2000 dilution) and anti-V-ATPase antiserum (1:5000) were used as primary antibodies. The primary antibodies were applied to the sections and incubated overnight at 4 °C. Subsequently, the sections were incubated with a mixture of goat anti-rabbit IgG labeled with Alexa Fluor 488 and goat anti-guinea pig IgG labeled with Alexa Fluor 555 (Molecular Probes, OR), both diluted 1:500 with PBS. The sections were incubated with secondary antibodies for 3 h at room temperature.

For next double immunofluorescence staining, sections were simultaneously incubated with anti-NKA antiserum (1:2000 diluted) and anti-NHE3 antiserum (1:2000 diluted), and then incubated with goat anti-rabbit IgG labeled with Alexa Fluor 488 and goat anti-guinea pig IgG labeled with Alexa Fluor 555 for 3 h at room temperature. The stained sections were observed under a confocal laser scanning microscope (LSM5 Pascal, Zeiss, Jena, Germany).

### Three-dimensional reconstruction

Amira software (version 5.4.1, Mercury Computer Systems, Berlin, Germany) was used for three-dimensional reconstruction of the follicular ionocytes in the gills of catshark embryos. The gills were dissected out from the fixed embryo at stage 33 and were paraffin-embedded as described above. Serial sections were cut at 10 μm thickness and were immunochemically stained with the antiserum specific for NKA or NHE3 by the ABC method in combination with a DAB labeling. Digital photographs of gill structures and follicular ionocytes taken with Keyence BZ-9000 microscope (Keyence Corporation, Osaka, Japan) were entered sequentially into Amira and aligned using the sum of least squares alignment algorithm. Additional alignment corrections were made visually by using the outlines of the NKA- and NHE3-positive follicular ionocytes as landmarks.

### Whole-mount double fluorescence immunohistochemistry

To further clarify the distribution of follicular and single ionocytes on the gills, we applied whole-mount immunohistochemistry with anti-NKA and anti-NHE3 antisera. For tissue clearing, the fixed gills of catshark embryos at stage 33 were immersed in scale CUBIC-1 solution (Fujifilm-Wako, Osaka, Japan) at 37 °C with gentle shaking for 3 days to remove lipids. After washing in PBS, gill samples were incubated with 5% NGS for 30 min at room temperature, and were sequentially incubated with a mixture of anti-NKA and anti-NHE3 antisera for 3 days at 37 °C. Both antisera were diluted with PBST (0.2% Triton X-100 in PBS) containing 5% NGS to a final dilution of 1:2000. The samples were then incubated for 2 days at 37 °C with a mixture of goat anti-rabbit IgG labeled with Alexa Fluor 488 and goat anti-guinea pig IgG labeled with Alexa Fluor 555 (Molecular Probes), both diluted 1:500 with PBST. Finally, the samples were immersed in Scale CUBIC-2 reagent overnight at 37 °C to be sufficiently transparent. The stained samples were observed under the confocal laser scanning microscope (LSM5 Pascal, Zeiss).

### Detection of extrabranchial ionocytes in catshark embryo

The embryos of stages 31 were examined for observation of ionocytes on the body skin. The fixed embryos were incubated with 3% H_2_O_2_ for 30 min and treated with 5% NGS in PBST for 30 min. The embryos were then incubated with anti-NKA antibody overnight at 4 °C. Subsequently, the samples were incubated with biotinylated anti-rabbit IgG for 30 min, and then with ABC for 30 min. Finally, the reaction products were visualized by using chromogen or DAB to yield blue or brown precipitate. The precipitation was detected under the light microscope (Eclipse Ci-L, Nikon). The head region of stained embryos was rinsed in PBS, placed in PBS containing 30% sucrose, and then frozen in Tissue-Tek OCT Compound (Sakura Finetek). Cryosections (20 μm thick) were cut in a cryostat (Leica) at −20 °C and mounted on glass slides (Matsunami Glass). The cross sections were observed under the light microscope. As a preliminary experiment, we also used anti-V-ATPase antibody to detect B-type ionocytes on the body skin of catshark embryo at stage 31. V-ATPase-positive cells were detected around the eyes and mouth (Fig. S1), and the distribution pattern was similar to that of the lateral line canal system in the head of the bonnethead shark, *Sphyrna tiburo* (Maruska 2001). Because we could not confirm the presence of V-ATPase-positive ionocytes on the skin, it is considered that major population of ionocytes on the embryonic skin is NKA-positive type-A cell.

For scanning electron microscopy, the embryos were fixed in 2% PFA-2% glutaraldehyde in PB. The fixed embryos were dehydrated in ethanol, immersed in t-butylalcohol, and freeze-dried. Dried samples were mounted on specimen stubs, coated with platinum palladium in an ion sputter (Hitachi E-1030, Tokyo, Japan), and examined with a Hitachi S-4800 scanning electron microscope.

Because fixed yolk is fragile, we used vital staining with DASPEI (2-(4-(dimethylamino)styryl)-*N*-ethylpyridinium iodide, Sigma) for the observation of ionocytes on the yolk-sac membrane. DASPEI is a mitochondrial vital probe which has been used to identify ionocytes in teleost fish (Bereiter-Hahn 1976; Karnaky et al. 1984). The stock solution of DASPEI (10 mM) was diluted 1:9 with seawater for detection of ionocytes, and then catshark embryo at stage 31 were incubated for 4 h. The mitochondria-rich ionocytes on the yolk-sac membrane and body skin of the head region were observed under the Keyence BZ-9000 microscope.

## Results

### Gill formation and ionocyte distribution during development

**Fig. 1:**
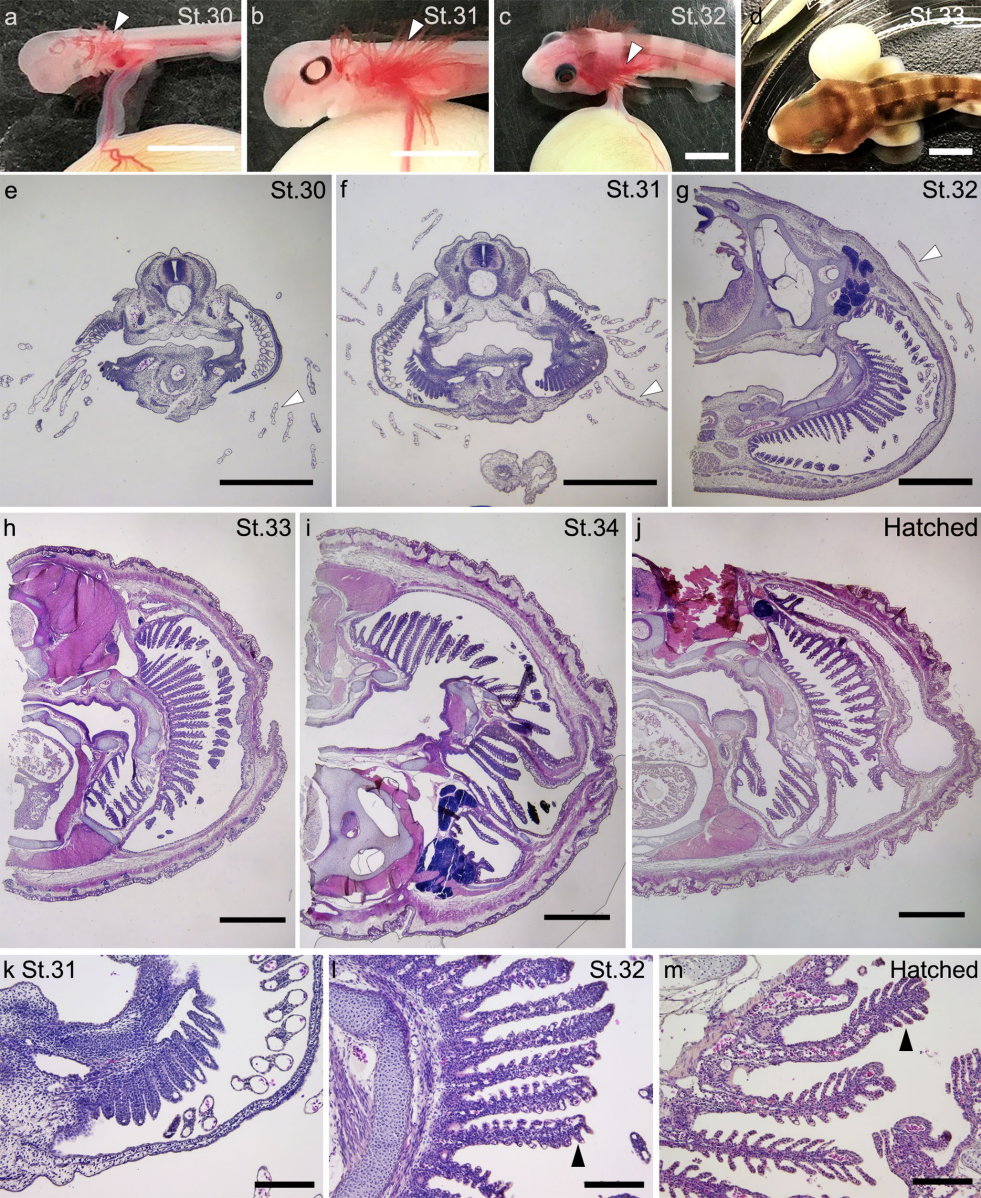
Developing gill of the cloudy catshark embryo. Developmental stages were identified according to Ballard et al. (1993). The head region focusing on external gills of catshark embryo at stage 30 (**a**), 31 (**b**), 32 (**c**), and 33 (**d**). Cross sections of the head including gills at stage 30 (**e**), 31 (**f**, **k**), 32 (**g**, **l**), 33(**h**), 34 (**i**), and hatched fish (**j**, **m**) with hematoxylin and eosin staining. Gill filaments increase in length, while external gill filaments (open arrowheads) are observed until stage 32. Gill lamellae (filled arrowheads) started appearing at stage 32. Scale bars: 5 mm (**a**–**d**), 1 mm (**e**–**j**), 200 μm (**k**–**m**)

Embryos were staged according to the developmental stages characterized in cloudy catshark (Takagi et al. 2017) based on those in lesser spotted dogfish (Ballard et al. 1993). The developmental patterns of the gill structure were examined by HE staining. At stage 30, the earliest stage examined in this study, external gills, internal gill septa, and filaments were already recognizable prior to the prehatching stage (Fig. 1a, e). At stage 31, the external gills and internal gill filaments were elongated, but the secondary lamellae had not yet developed (Fig. 1b, f, k). As shown in previous studies (Ballard et al. 1993; Takagi et al. 2017), the external gill filaments seem to reach the maximum length at stage 32 (Fig. 1c), and secondary lamellae appeared on the extended filaments in the internal gills (Fig. 1g, l). However, the lamellae were still poorly developed, and the surface area of gills was smaller than that in the later stages (Fig. 1l). At stages 33 and 34, whereas the external gills diminished (Fig. 1d), gill lamellae extended from both sides of the gill filaments which appeared equal to those of hatched juveniles (Fig. 1h–j and m).

**Fig. 2:**
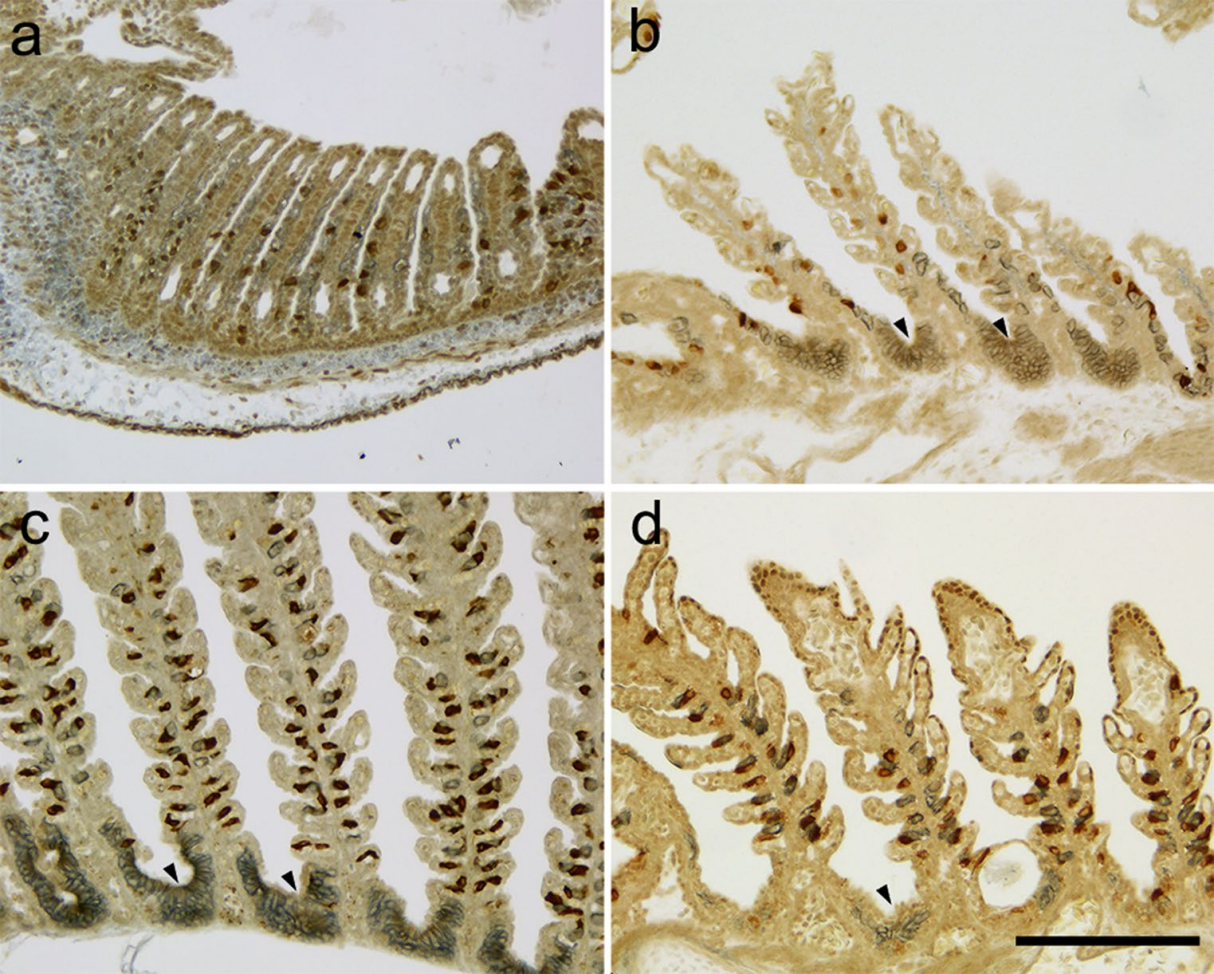
Ionocytes on the gills of the cloudy catshark embryo. Cross sections of gills in embryos at stage 31 (**a**), 32 (**b**), 34 (**c**), and hatched fish (**d**) immunohistochemically stained with anti-Na^+^/K^+^-ATPase (blue) and anti-V-ATPase (brown). Immunoreactive ionocytes first appeared in the gills at stage 31 and follicular ionocytes (arrowheads) are detected from stage 32. Scale bar: 200 μm

The timing of occurrence of gill ionocytes was examined by double-colored immunohistochemistry with anti-NKA and anti-V-ATPase antibodies. Both NKA- and V-ATPase-immunoreactive ionocytes in the gills first appeared at stage 31, and their distribution is restricted to the gill filaments (Fig. 2a). Neither NKA- nor V-ATPase-immunoreactive ionocytes was found in the external gills. At stage 32, in addition to NKA- and V-ATPase-immunoreactive ionocytes distinctly observed in the gill filaments, NKA-immunoreactive ionocytes in the gill septa were arranged in a radial fashion, forming sac-like structure (Fig. 2b). The follicular structure of NKA-rich ionocytes was developed in embryos at stage 32–34 and in hatched fish (Fig. 2b–d).

### Localization of NKA, V-ATPase, and NHE3 in embryonic gills

**Fig. 3:**
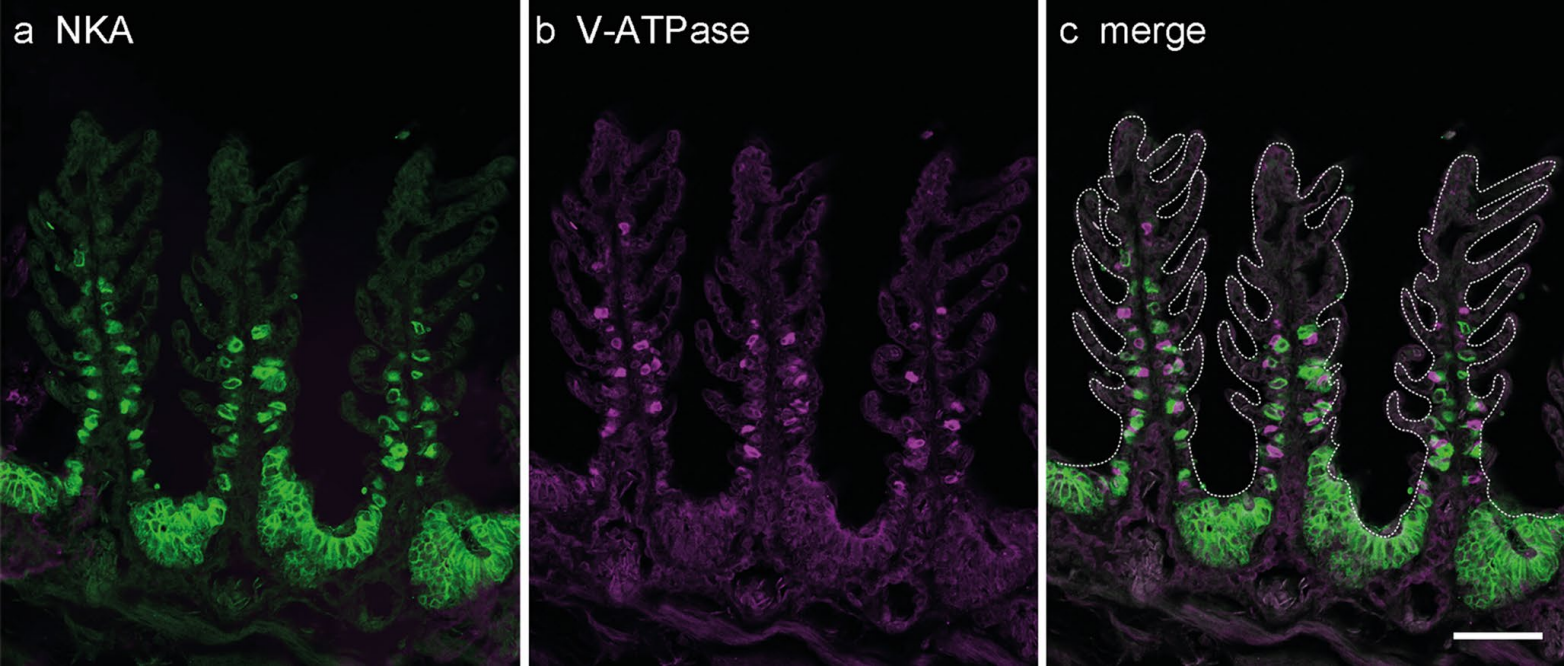
Immunolocalization of Na^+^/K^+^-ATPase (NKA) and V-ATPase in the gills of the cloudy catshark embryo. Cross sections of gills in embryos at stage 32 were immunohistochemically stained with anti-NKA (green, **a**) and anti-V-ATPase (magenta, **b**). **c** Merged image. NKA immunosignals were detected in both single ionocytes in the gill filaments and follicular cells in the gill septum. V-ATPase signals were detected only in single ionocytes of filaments. Scale bars: 100 μm

To examine the localization of type-A and type-B ionocytes in embryonic gills, the localization of NKA and V-ATPase was detected in the cross section of the catshark gills at stage 32 by double immunofluorescent staining (Fig. 3). The NKA-rich cells were observed as solitary cells in the gill filaments and as a follicular structure in the gill septa. The solitary NKA-rich cells have a small rectangular shape (average 31.5 μm width, SD 3.5 μm, *n* = 9), and their immunosignals are labeled over the cell with the nucleus remaining unstained. The immunosignals in follicular NKA-rich ionocytes were found only at the edge of cells on the basolateral side. The follicular structure typically consisted of multiple NKA-rich ionocytes, (average 141.0 μm width, SD 23.4 μm, *n* = 9) (Fig. 3a). On the other hand, V-ATPase-rich cells (average 21.9 μm width, SD 3.4 μm, *n* = 9) are detected only in the gill filaments as solitary cells. As the solitary NKA-rich cells, V-ATPase-immunosignals were also detectable throughout the cells except the nuclei (Fig. 3b). Immunoreaction for V-ATPase was not detected in NKA-rich cells but often adjacent to NKA-rich cells (Fig. 3c). The number of solitary NKA- and V-ATPase-rich cells in one filament was 5.3 ± 3.2 and 3.8 ± 1.3 (average ± SD), respectively, whereas each gill filament possess one follicular ionocyte.

**Fig. 4:**
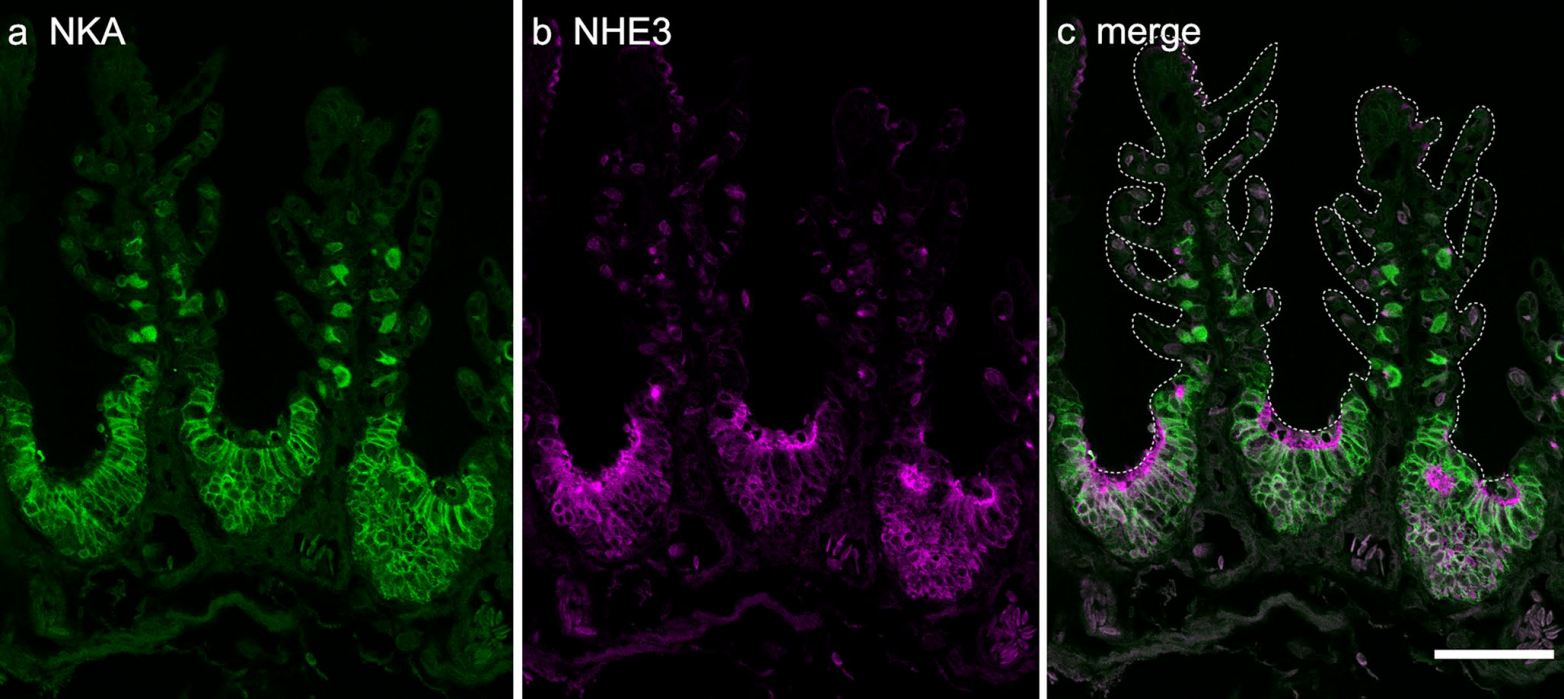
Immunolocalization of Na^+^/K^+^-ATPase (NKA) and Na^+^/H^+^ exchanger 3 (NHE3) in the gills of the cloudy catshark embryo. Cross sections of gills at stage 33 were immunohistochemically stained with anti-NKA (green, **a**) and anti-NHE3 (magenta, **b**). **c** Merged image. Both of basolateral NKA and apical NHE3 were localized in the same cells and detected in both single ionocytes in the gill filaments and follicular cells in the gill septum. Scale bars: 100 μm

Next, to understand the function of follicular NKA-rich ionocytes in the embryonic gills, NKA and NHE3 were detected by double immunofluorescent staining. The NHE3 immunoreactivity was detected in the apical membrane of both solitary and follicular NKA-rich ionocytes. The NHE3-positive apical region is different in morphology between single and follicular NKA-rich cells (Fig. 4a, b). The single NKA-rich cells showed relatively small NHE3 immunoreactivity in the apical region. In follicular NKA-rich cells, the NHE3-immunoreactive apical region was enlarged and shared a common apical crypt with other NKA-rich cells in the same aggregate (Fig. 4c).

### Three-dimensional structure of follicular NKA-rich ionocyte

**Fig. 5:**
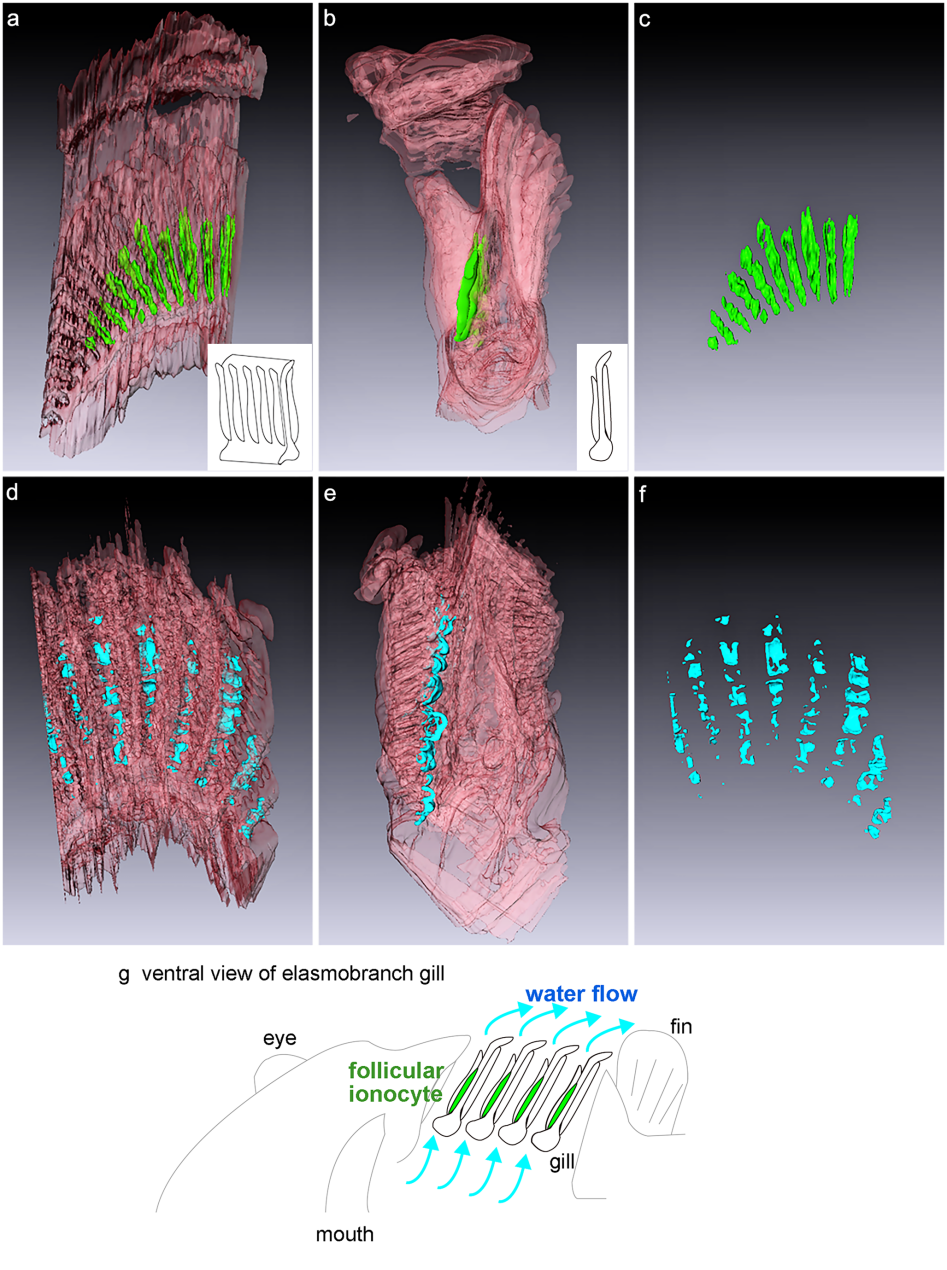
Three-dimensional (3D) structure of follicular ionocytes in the gills of the cloudy cat shark embryo. 3D reconstructions were generated from paraffin section of gills immunohistochemically stained with anti-Na^+^/K^+^-ATPase (NKA, light green, **a**–**c**) and anti-Na^+^/H^+^ exchanger 3 (NHE3, light blue, **d**–**f**) of follicular ionocytes. **a**, **d** Frontal view of immunosignals in the whole gills. **b**, **e** Lateral view of immunosignals in the whole gills. **c**, **f** Frontal view of NKA and NHE3 signals. **g** Schematic diagram showing the distribution of follicular ionocytes in the rostral sides of the gill septum and water flow through the gills

Three-dimensional reconstruction reveals that catshark embryo at stage 33 has a developed gill structure like that of adult fish. The frontal views of whole gills show the interbranchial septum extending from gill arch to the lateral edge of the body to form gill slits (Fig. 5a, d). From the lateral view of whole gills, rostral- and caudal-side gill filaments radiate from both sides of the gill septa (Fig. 5b, e). To examine the 3D structure and distribution of follicular NKA-rich ionocytes, 3D reconstructions of NKA and NHE3 immunoreactivity were generated from follicular ionocytes in the gill septum. The follicular structures of NKA-rich cells are localized at the proximal part of the gills, and their distribution is limited to the rostral side of the gill septum (Fig. 5a, b, g). These cells form a peapod-like structure in each inter-filament region of the gill septum (Fig. 5c). The immunoreactivity of NHE3 appears only at the rostral side of the gill septum as NKA; however, the 3D structure of the immunosignals is different between NKA and NHE3 (Fig. 5d, e). While NKA signals form one elongated aggregates in the inter-filament space, the distribution of NHE3 signals shows multiple U-shaped structures in line (Fig. 5e, f).

### Localization of NKA and NHE3 on the rostral and caudal sides of gill septum

**Fig. 6:**
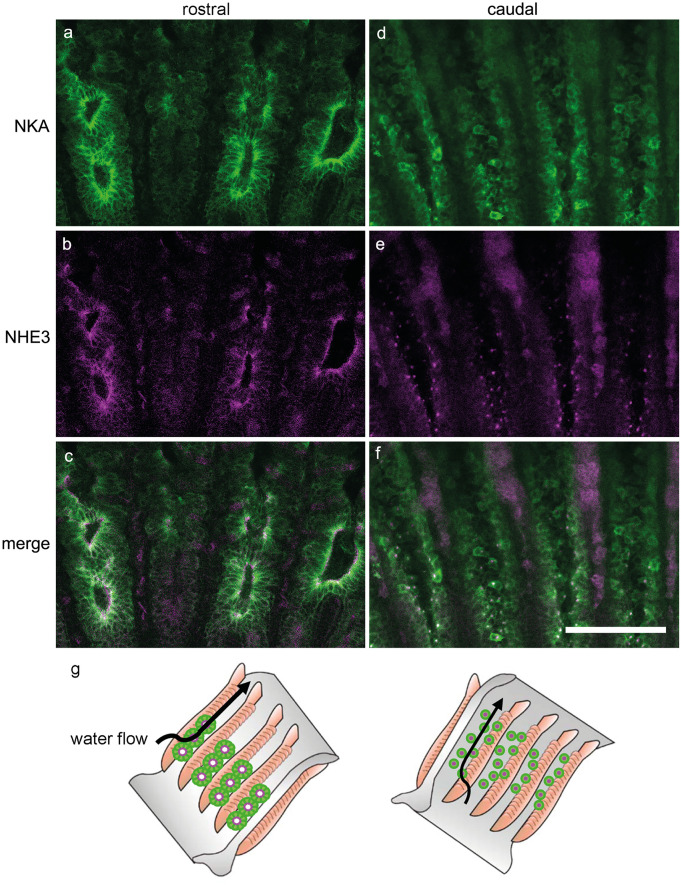
Whole mount immunostaining for Na^+^/K^+^-ATPase (NKA) and Na^+^/H^+^ exchanger 3 (NHE3) in the gills of the cloudy catshark embryo. Double immunofluorescence staining with anti-NKA (**a** and **d**, green) and anti-NHE3 antisera (**b** and **e**, magenta) in the gills of catshark embryo at stage 33. **c**, **f** Merged image. Follicular structure of ionocytes were observed in the gill septum of rostral side (**a**–**c**), but not detected in the septum of caudal side (**d**–**f**). **g** Schematic diagrams showing the distribution of follicular and single NKA-immupositive ionocytes on the rostral and caudal side of the gill septum. Scale bar: 200 μm

To further reveal the localization and structure of follicular NKA-rich ionocytes in the gill septum, a whole-mount preparation of the cleared gill at stage 33 was stained with anti-NKA and anti-NHE3 antibodies and observed by confocal laser scanning microscopy. In the rostral side of the gill septum, NHE3 immunoreaction was detected in the apical region of follicular NKA-rich ionocytes and located in line (Fig. 6a–c). On the other hand, the caudal side of the gill septum has only solitary NKA rich ionocytes with small apical signals of NHE3 (Fig. 6d–f). Since one NKA-rich aggregate has several NHE-positive spots, the distribution of NKA and NHE3 immunoreactivity is like pea-and-pod relationship (Fig. 6g).

### Extrabranchial ionocytes of catshark embryo

**Fig. 7:**
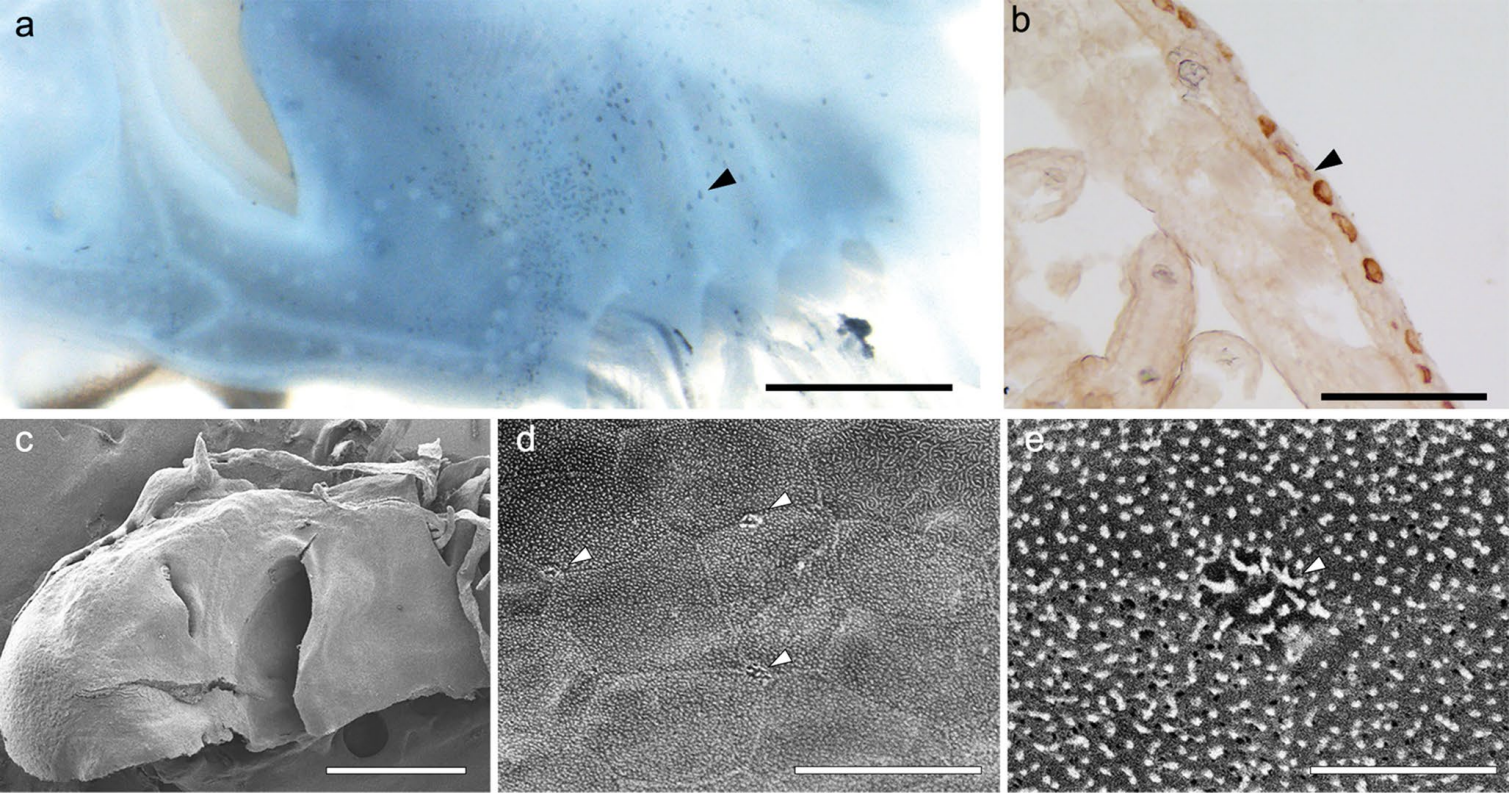
Ionocytes on the embryonic skin. Whole-mount immunohistochemistry for Na^+^/K^+^-ATPase (NKA, blue) of the skin around the jaw (**a**). The cross section of head region immunohistochemically stained with anti-NKA (brown, **b**). NKA-rich ionocytes (filled arrowheads) are distributed on the skin epithelium outside of internal gills. Scanning electron micrographs of skin in the head region (**c**) and skin around jaw (**d**, **e**) of catshark embryo. The apical openings were equipped with short microvilli (open arrowheads). Scale bars: 1 mm (**a**, **c**), 100 μm (**b**), 20 μm (**d**), 5 μm (**e**)

**Fig. 8:**
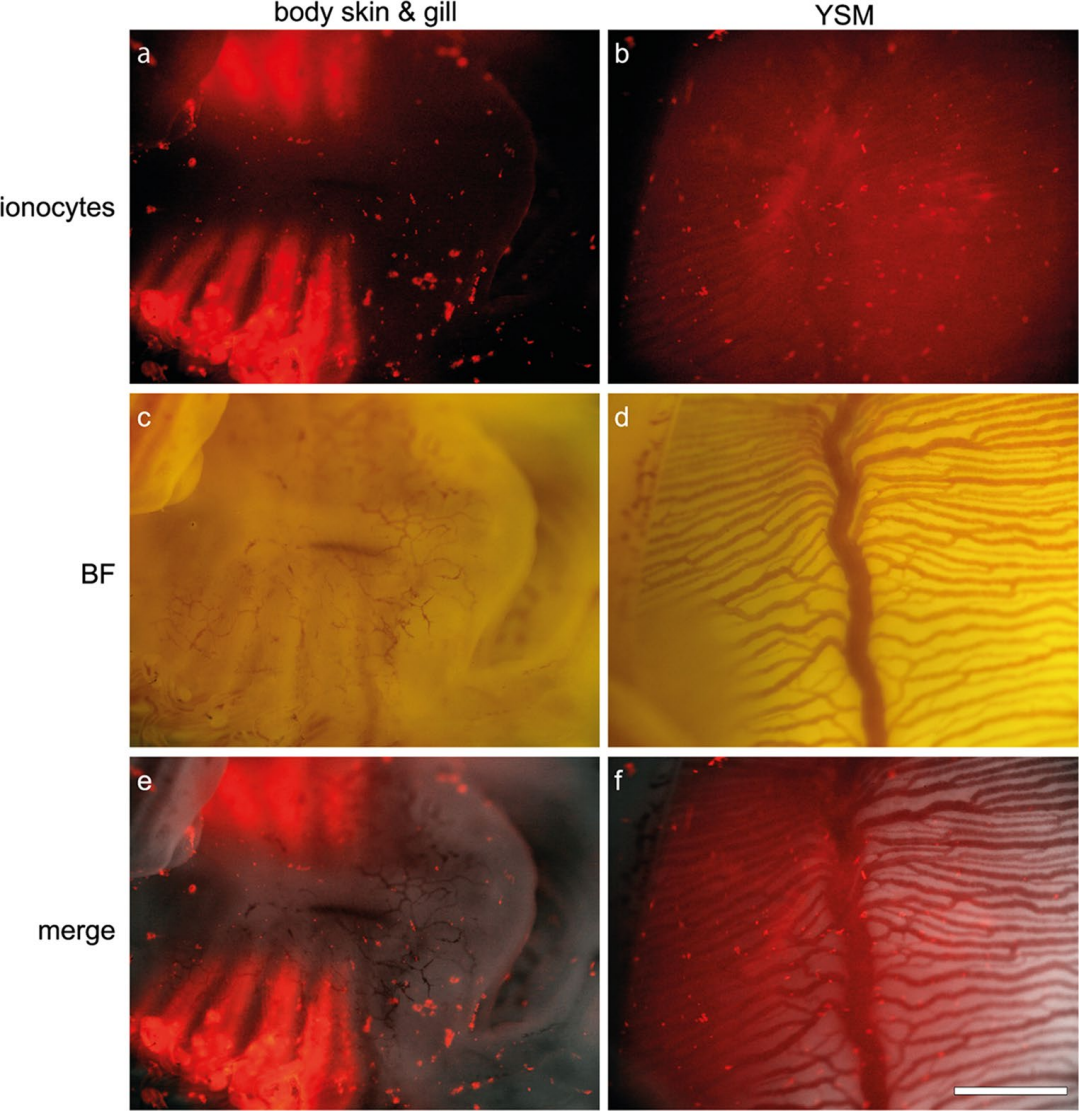
Ionocytes on the embryonic skin and yolk-sac membrane (YSM). DASPEI-positive ionocytes (**a**, **b**) and bright field microscopic images of same region (**c**, **d**). **e**, **f** Merged image. DASPEI-positive ionocytes are detected on body skin around under jaw, gills (**a**, **c**, **e**) and YSM (**b**, **d**, **f**). Scale bar: 1 mm

To examine the extrabranchial ionocytes before the gills are fully functional, we observed type-A ionocytes on the body skin of catshark embryos by immunohistochemistry with anti-NKA antiserum. Because the NKA-immunopositive type-A ionocytes on the body skin were observed most clearly at stage 31 by whole-mount immunohistochemistry, the embryos at this stage were used for further observation. The embryos at stage 31 have high density of NKA-rich ionocytes on the body skin especially around the jaw (Fig. 7a). By observing cross cryosections of their head region, we found solitary NKA-rich ionocytes on the skin epithelium outside of the internal gills (Fig. 7b). Scanning electron microscopic observation also provides evidence that ionocytes on the embryonic body skin are in direct contact with the external environment through their apical membrane. The apical openings of ionocytes were found on the lower jaw (Fig. 7c, d). The apical openings were equipped with short microvilli, and their size is relatively small, approximately 1 μm (Fig. 7e). We attempted to detect the NKA-rich ionocytes on the yolk-sac membrane by immunohistochemistry, but the fixed yolk-sac membrane was fragile and immunosignals were difficult to distinguish from artifacts. Instead of this method, the ionocytes of the yolk-sac membrane and body skin were labeled with a mitochondrial probe DASPEI in living embryos at stage 31 (Fig. 8). This technique does not distinguish whether these cells are NKA-rich or V-ATPase-rich ionocytes. The mitochondria-rich ionocytes of the head region were distributed on the body skin of the lower jaw and internal gills, similar to the results of immunohistochemistry (Fig. 8a, c, e). The mitochondria-rich ionocytes were also observed on the yolk-sac membrane. The ionocytes are abundant near blood vessels of the yolk-sac membrane (Fig. 8b, d, f).

## Discussion

In the present study, we described the development of gill ionocytes in the catshark embryo. The catshark embryo at stage 31 developed two types of single ionocytes, NKA-rich and V-ATPase rich ionocytes, in the gill filaments, while NKA-rich ionocytes were also observed on body skin and yolk-sac membrane. At stage 32, in addition to single ionocytes on the gill filaments, a follicular structure of ionocytes is developed in the gill septa. This follicular ionocytes shows NKA in the basolateral membrane and NHE3 in the apical membrane, indicating the function of Na^+^ uptake and H^+^ secretion. Three-dimensional analysis revealed that their distribution was limited to the rostral side of the gill septum, which might facilitate ion transport between external environment and body fluid in the embryo before the gills were fully developed.

It has been reported that the external gills are an important respiratory organ in cartilaginous embryos (Baranes and Wendling 1981; Hamlett et al. 1985). In this study, ionocyte was not detected in the external gills at all stages examined. This result indicates that the external gill is not involved in ion regulation at least after stage 30. In catshark embryos, the external gills reached the maximum length at stage 32, while pre-hatching occurs during stage 31 (Takagi et al. 2017). After pre-hatching, the embryo starts buccal pumping for respiration to pass water over the developed internal gills (Tomita et al. 2014). These findings indicate that the primary site of respiration in catshark embryo shifted from the external gills to the internal gills located inside the gill slit after pre-hatching. The respiratory function of external and internal gills has been mentioned in some studies (Hamlett et al. 1985; Pelster and Bemis 1992; Leonard et al. 1999; Tomita et al. 2014); however, to our knowledge, the gill ionoregulatory function in cartilaginous embryos has not been reported to date.

The gill filaments inside the gill slits were already recognizable at stage 30, but ionocyte was not yet detected. At stage 31, by means of immunohistochemistry with the antisera specific for NKA and V-ATPase, both type-A and type-B ionocytes appeared on the gill filaments (Fig. 2a). Pre-hatching event occurs in the middle of this stage. It has been reported that pre-hatching is a physiological turning point for cartilaginous fish development. Honda et al. (2020) showed that embryos began to incorporate yolk into the intestine and absorb nutrition from their yolk sac after pre-hatching, and the nutritional absorption in the intestine of catshark embryo was different between before and after the pre-hatching. We also compared the gill sections between prior and following the pre-hatching, but no drastic change in gill filament morphology and ionocyte distribution was recognized during stage 31. In teleost species, it has been reported that ionocytes first appear in the yolk-sac membrane in the early embryonic stage, followed by their appearance in the body skin in the late embryonic stages, and then shifts to the gills and opercular membrane in larval and later developmental stages (Kaneko et al. 2008). In cartilaginous fishes, the yolk-sac membrane contributes to urea homeostasis until the liver and other extrahepatic organs become fully functional (Takagi et al. 2014, 2017), indicating the important role of yolk-sac membrane on osmoregulation. In the present study, the mitochondria-rich ionocytes were observed also on the yolk-sac membrane and body skin of catshark embryo at stage 31 (Fig. 8). On the yolk-sac membrane, ionocytes were mainly distributed along blood vessels. The embryo of teleost fish, medaka (*Oryzias latipes*) also showed that their ionocytes on the yolk-sac membrane contact blood vessels (Miyanishi et al. 2013). This distribution pattern of ionocytes is beneficial for facilitating ion transport between blood and aquatic environments. The NKA-immunoreactive ionocytes were densely distributed also on the body skin, and their apical membrane is equipped with microvilli (Fig. 7). Because it was reported that microvilli were generally seen in all ionocytes in the gills of dog fish (*Squalus acanthias*) (Wilson et al. 2002), our findings suggest that ionocytes on the skin is as functional as those on the gill filaments in the catshark embryo at stage 31. The gill lamellae started to develop at stage 32, when NKA- and V-ATPase-immunoreaction was clearly detected in the ionocytes on the gill filaments (Fig. 2b). It was reported that catshark embryos start buccal pumping to take waters in through the mouth and expel it through the gill slits after pre-hatching, presumably at stage 31 or 32 (Tomita et al. 2014). Water flow through the gills is considered to contribute not only to respiration but also to ion regulation. After stage 32, the gill secondary lamellae enlarged their surface area, and numerous solitary ionocytes are constantly detected. In addition to solitary ioncytes on the gill filaments, ionocytes form follicular structure on the gill septum. This rapid development of branchial ionocytes and water flow through the gills might indicate the shift of the ion and acid-base regulatory site from the body skin to the gills after prehatching.

Branchial ionocytes of elasmobranch species have been classified into two distinct types: type A and type B. In the present study, we detected type-A and type-B ionocytes in developing embryos by double fluorescent immunohistochemistry with the antisera specific for NKA and V-ATPase (Fig. 3). While solitary ionocytes in the gill filaments were immunopositive for either NKA or V-ATPase, the follicular ionocyte on the gill septum showed only NKA staining. This aggregated ionocyte is similar to “follicularly-arranged NKA-rich cells (follicular NRCs)” found in the gill septum of adult Japanese banded houndshark (Takabe et al. 2012). The follicular NRCs were found almost exclusively in the proximal region of the gills, and they expressed NHE3 in the apical membrane (Takabe et al. 2012). Similar to adult houndshark, the double fluorescent immunohistochemistry for NHE3 and NKA showed that apical NHE3 was observed not only in the solitary NKA-rich ionocytes but also in the follicular ionocytes in catshark embryo (Fig. 4). A similar follicular ionocytes have been reported in the gills of Osorezan dace inhabiting extremely acidic water (Kaneko et al. 1999). In the gills of Osorezan dace, ionocytes (chloride cells) are arranged in a radial fashion to form a follicular structure. Those ionocytes are reported to express NKA, NHE3, Na^+^, HCO_3_^−^ cotransporter 1 (NBC1), aquaporin 3 (AQP3), and carbonic anhydrase 2 (CA2) to excrete H^+^ and to retain HCO_3_^−^ for neutralization of plasma (Hirata et al. 2003). The follicular structures of ionocytes are considered to contribute to increase total apical surface area of ionocytes without reducing the gill respiratory surface (Kaneko et al. 1999). The follicular NRCs of Japanese banded houndshark also possessed basolateral NKA/apical NHE3 and expressed CA2 mRNA, indicating that the follicular NRCs may be involved in acid-base regulation similar to the follicular ionocytes in the Osorezan dace (Hirata et al. 2003; Takabe et al. 2012). Together, the follicular NKA-rich ionocytes commonly present in teleost and cartilaginous fish, and they might contribute to acid-base regulation also in catshark embryos after prehatching, when the external gill gradually shortened and the respiratory area of the internal gill was in process of development.

For acid-base regulation and other branchial functions, the water typically enters the pharynx via mouth, and then pass over the gill surface. In elasmobranch gills, the ingested water first passes through interlamellar channels and encounters the inter-branchial septum (Wegner 2015). In the present study, the three-dimensional structural analyses and whole-mount immunohistochemistry revealed that multiple follicular ionocytes were localized in line on the proximal part of the gill septum, which corresponds to the major pathway of water. Moreover, the distribution of follicular ionocytes was limited to the rostral side of the gill septum (Figs. 5 and 6). The characteristics of single and follicular NKA-rich ionocytes and their distribution are summarized in Fig. 6g. Since gill flaps gently curved backward, it was most likely that the rostral sides of the gill septum is exposed to faster water flow than the caudal side (Figs. 5g and 6g). In the gills of catshark embryos, the secondary lamellae of embryo are not fully elongated, suggesting their surface-area ratio of gills to the whole body is not as large as hatched larvae and adult fish. The follicular ionocytes on rostral side of the gill septum are considered to facilitate acid-base regulation in the embryo before the gills are fully developed. Besides, the gill epithelium primarily consists of pavement cells and ionocytes. Since pavement cells and ionocytes are assumed to be important for gas exchange and ion regulation, respectively, trade-offs occur between the two physiological events on the gill epithelium. Within the limited surface area of the gill epithelium, the efficient body fluid regulation by the follicular ionocytes contributes to balance with the respiratory function of pavement cells.

In summary, we have elucidated the early development of ionocytes in elasmobranch fishes. In common with teleosts, elasmobranch embryos have ionocytes on the yolk sac membrane and body skin. On the other hand, the follicular structure of the ionocytes appears on the gill septum from stage 32 when the respiratory area shifts from the external gills to the developing internal gills. The follicular ionocytes were only detected on the rostral side of the gill septum. This dissymmetric distribution of follicular ionocytes can be interpreted as an ion regulation strategy that allows efficient ion regulation by using the water flow generated by the gill septum, which is unique to cartilaginous fish.

### Supplementary Information

Below is the link to the electronic supplementary material.

Supplementary file1 (PDF 266 KB)

## Data Availability

All data supporting the findings of this study are available within the paper and its Supplementary Information.
