# Supplementary material for: Development of branchial ionocytes in embryonic and larval stages of cloudy catshark, Scyliorhinus torazame
Source: Cell Tissue Res. 2024 May 15;397(2):81–95. doi: 10.1007/s00441-024-03897-4 (PMC11291541; doi:10.1007/s00441-024-03897-4)
Supplement: Supplementary file 1 — Supplementary file1 (PDF 266 KB) [file 441_2024_3897_MOESM1_ESM.pdf]

Title: Development of branchial ionocytes in embryonic and larval stages of cloudy catshark, *Scyliorhinus torazame*

Journal name: Cell and Tissue Research

Author names: Mayu Inokuchi, Yumiko Someya, Keitaro Endo, Katsunori Kamioka, Wataru Katano, Wataru Takagi, Yuki Honda, Nobuhiro Ogawa, Kazuko Koshiba-Takeuchi, Ritsuko Ohtani-Kaneko, Susumu Hyodo

Corresponding author:
Mayu Inokuchi
Department of Aquatic Bioscience, Graduate School of Agricultural and Life Sciences, The University of Tokyo
inokuchimayu@g.ecc.u-tokyo.ac.jp

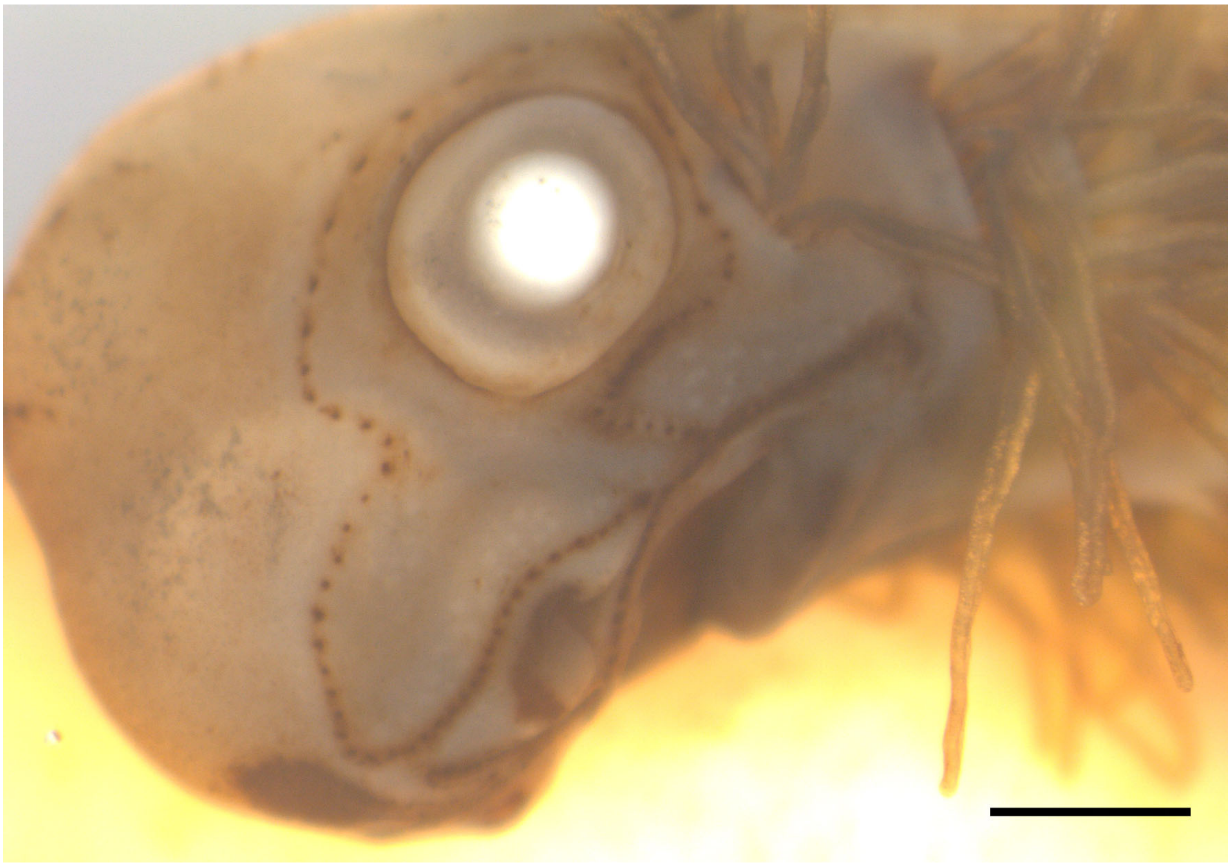

Fig. S1 V-ATPase-positive cells (brown) on the body skin of a catshark embryo at stage 31. V-ATPase-positive cells are detected around the eyes and mouth, but the distribution pattern is similar to that of the lateral line canal system. The presence of V-ATPase-positive ionocytes was not confirmed on the skin. Scale bar: 1 mm.
